# Laboratory Determination vs. Clinical Judgment in Controlling Wound Closure

**Published:** 1918-12

**Authors:** 


### LABORATORY DETERMINATION VS. CLINICAL JUDGMENT IN CONTROLLING WOUND CLOSURE

Evidence is accumulating that the bacteriologic wound standard for closure, so admirably worked out by Carrel for the hypochlorites, may not hold for the bacterial survivals of bichlorid; the formula for the bacterial survivals of bichlorid may not hold for the survivors of carbolic; nor the carbolic habitues for flavine; nor the flavinites for bip. Perhaps owing to the power of biologic adaptation each antiseptic must have its own bacterial standard for closure. Each antiseptic may require a separate index number to express the amount of injury to the living tissue, and to express the heightening of bacterial virulence.

It may be possible that a wound that has had no antiseptic, hence, no high-bred virulence, no tissue injury, may be closed with numerous bacteria; while the wound whose tissue has been damaged continuously by an antiseptic, and in addition whose inhabiting bacteria have gone through a selective stepping up in virulence by the antagonism of an antiseptic, cannot be closed with an equal number of bacteria. The student of surgery soon learns to " read the wound " with accuracy; not only the wound, but the patient; not only the patient, but the environment. The bacteriologist has no criterion by which he can say that the patient is enfeebled, that hence his power of wound healing is low, and that on that ground suture is not advisable. The surgeon has. The bacteriologist sees one side — the bacteria. The clinician and the bacteriologist are both making biologic observations — but the clinician is observing the effect of the contest between the bacteria and the host.

